# Comparative analysis of adipose-, bone marrow-, and amniotic membrane-derived MSC secretomes and EVs reveals shared and source-specific therapeutic signatures for osteoarthritis

**DOI:** 10.20517/evcna.2025.115

**Published:** 2025-12-30

**Authors:** Enrico Ragni, Andrea Papait, Michela Maria Taiana, Paola De Luca, Giulio Grieco, Elsa Vertua, Pietro Romele, Antonietta Rosa Silini, Ornella Parolini, Laura de Girolamo

**Affiliations:** ^1^IRCCS Ospedale Galeazzi - Sant’Ambrogio, Laboratorio di Biotecnologie Applicate all’Ortopedia, Milano 20157, Italy.; ^2^Dipartimento di Scienze della Vita e Sanità Pubblica, Università Cattolica del Sacro Cuore, Roma 00168, Italy.; ^3^Fondazione Policlinico Universitario A. Gemelli IRCCS, Roma 00168, Italy.; ^4^Centro di Ricerca E. Menni, Fondazione Poliambulanza Istituto Ospedaliero, Brescia 25124, Italy.; ^5^Fondazione IRCCS Casa Sollievo della Sofferenza, San Giovanni Rotondo 71013, Italy.

**Keywords:** Mesenchymal stromal cells, secretome, extracellular vesicles, miRNAs, osteoarthritis, chondroprotection, paracrine signaling, tissue regeneration

## Abstract

**Aim:** Mesenchymal stromal cells (MSCs) exert their therapeutic effects in osteoarthritis (OA) primarily through paracrine signaling, including secreted proteins and extracellular vesicle (EV)-associated microRNAs (miRNAs). However, the contribution of tissue origin to the composition and function of these secretomes remains unclear. This study aimed to provide a comprehensive molecular and functional comparison of secretomes from adipose-derived (ASCs), bone marrow-derived MSCs (BMSCs) and human amniotic membrane-derived MSCs, with a specific focus on OA-relevant pathways.

**Methods:** MSCs were immunophenotyped by flow cytometry. Secretomes were profiled for 200 factors and 784 EV-miRNAs. Functional enrichment was performed using Gene Ontology and Reactome databases. *In vitro*, secretomes were tested on interleukin (IL)-1β-stimulated human chondrocytes to assess modulation of OA-related gene expression.

**Results:** All MSC secretomes shared a core of factors enriched in anti-inflammatory and matrix-regulatory functions. ASCs showed the differential expression of a few modulators, potentially shifting their chondroprotective phenotype. EV-miRNAs further distinguished the MSC types. ASCs and BMSCs clustered closely in both overall miRNA content and functional enrichment, which included pathways for extracellular matrix organization, angiogenesis and IL-6 signaling. BMSC- and ASC-EVs had a higher ratio of OA-protective to destructive miRNAs, including miR-24-3p, miR-125b-5p and miR-222-3p. Functional assays confirmed that all MSC secretomes were effective in suppressing key OA-related genes in inflamed chondrocytes, with ASCs and BMSCs having a stronger activity.

**Conclusion:** These findings support the development of MSC-derived cell-free therapies and emphasize the importance of molecular profiling in MSC source selection. Further studies are warranted to validate these observations and optimize MSC-based interventions for clinical translation in OA.

## INTRODUCTION

Osteoarthritis (OA) is a prevalent and debilitating joint disease characterized by progressive degeneration of articular cartilage, subchondral bone remodeling, synovial inflammation and chronic pain, ultimately impairing mobility and quality of life^[1]^. Despite the high burden of disease, current therapeutic strategies are largely palliative, aimed at symptom relief rather than modifying disease progression^[2]^. In recent years, regenerative medicine approaches have emerged as promising alternatives, particularly those involving injections of mesenchymal stromal cells (MSCs)^[3]^. Among these, one-step procedures using MSC-enriched products derived intraoperatively from sources such as adipose tissue (stromal vascular fraction or microfragmented adipose tissue)^[4]^, bone marrow (bone marrow aspirate concentrate)^[5]^ or amniotic membrane (tissue or suspension)^[6]^ are gaining traction for their feasibility, minimal manipulation and immediate clinical applicability. These point-of-care strategies offer the advantage of bypassing *ex vivo* expansion^[7]^, aligning with regulatory guidelines for minimally manipulated tissue products while aiming to harness the therapeutic potential of MSCs in a single surgical session^[8]^.

MSCs, especially when injected into the joint cavity, exert their therapeutic effects primarily through paracrine mechanisms rather than direct tissue integration or differentiation. Upon administration, they release a complex repertoire of bioactive molecules, including cytokines, growth factors and chemokines, that modulate the local immune response, inhibit inflammation and support tissue repair^[9]^. A key component of this paracrine activity is the secretion of extracellular vesicles (EVs), nanoscale particles that carry proteins, lipids, and nucleic acids - including microRNAs (miRNAs) - which can be taken up by recipient cells within the joint environment^[10]^. These EV-associated miRNAs play a central role in regulating gene expression in target cells, influencing processes such as cartilage matrix metabolism and synovial inflammation. For instance, certain miRNAs carried by MSC-derived EVs have been shown to suppress catabolic enzymes and pro-inflammatory mediators, while promoting anabolic pathways and chondrocyte survival^[11]^. This indirect, cell-free mode of action is increasingly recognized as a critical contributor to the regenerative potential of MSC-based therapies in OA.

Notably, the composition and potency of MSC-derived factors vary significantly depending on the tissue of origin^[12]^. Adipose-derived MSCs (ASCs) are often enriched in anti-inflammatory cytokines and pro-regenerative growth factors^[13,14]^, while bone marrow-derived MSCs (BMSCs) may produce higher levels of angiogenic and osteoinductive signals^[15,16]^. Likewise, amniotic-derived MSCs [human amniotic membrane-derived MSCs (hAMSCs)] have been reported to secrete a unique set of immunomodulatory molecules and EVs enriched in miRNAs involved in tissue protection and immune regulation^[17,18]^. Understanding these source-dependent variations is critical for moving beyond a “one-size-fits-all” approach and towards precision therapies where cell-based approaches can be tailored to specific clinical needs. By systematically comparing the paracrine signatures, both factors and EV-associated miRNAs of ASCs, BMSCs and hAMSCs, the present study aims to identify distinctive molecular fingerprints that could guide the selection of the most suitable MSC source for OA treatment. Such knowledge will lay the foundation for more targeted and effective regenerative strategies, ultimately maximizing clinical benefit for patients.

## METHODS

### Ethics approval and consent to participate

The study was conducted in accordance with the Declaration of Helsinki and approved by the San Raffaele Hospital Ethics Committee (“Caratterizzazione e valutazione del potenziale rigenerativo delle cellule progenitrici tessuto specifiche ottenute da tessuti muscoloscheletrici”, approval on December 16, 2020, registered under number 214/int/2020 for surgical waste material) and by the Comitato Etico Provinciale di Brescia (Provincial Ethics Committee of Brescia) (“Studio delle applicazioni dei tessuti placentari e delle cellule da questi isolate in medicina rigenerativa,” approval on January 19, 2016, registered under number NP 2243 for hAMSCs). Informed consent was obtained from all subjects involved in the study.

### Human specimens’ collection and MSC isolation/expansion

Subcutaneous adipose tissue, purchased from Wepredic (Saint-Grégoire, France), was obtained from three healthy females (age 32 ± 6 years) undergoing aesthetic surgery procedures. Bone marrow was collected from three female donors (age 50 ± 2 years) undergoing iliac crest marrow aspiration. Human term placentae were collected from three healthy women after vaginal delivery or cesarean section at term. Isolation and expansion of ASCs, BMSCs, and hAMSCs were performed according to previously described protocols^[19-21]^. ASCs and BMSCs were expanded in Dulbecco’s Modified Eagle Medium F12 (DMEM/F12) supplemented with 10% Foetal Bovine Serum (FBS) (both from Thermo Fisher Scientific, Waltham, Massachusetts, USA), while hAMSCs were expanded in complete CHANG C medium (Irvine Scientific, Irvine, California, USA). All cultures were maintained at 37 °C, 5% CO2, and 95% humidity prior to secretome production and flow cytometry analysis. Cells at passages 2-3 were used for all experimental procedures to ensure consistency and preserve early-passage characteristics.

### Flow cytometry characterization of MSCs

MSCs at 90% confluence in the different culture media were detached and resuspended in Fluorescent-Activated Cell Sorter (FACS) buffer [1 × Phosphate-buffered saline (PBS), 2% FBS, 1 mM Ethylenediaminetetraacetic acid (EDTA)]. A total of 100,000 cells per condition were either left unstained or stained with fluorochrome-conjugated antibodies as follows: anti-CD45-PE-Vio770 (clone REA747), CD73-PE (clone REA804), and CD90-FITC (clone REA897) from Miltenyi Biotec (Bergisch Gladbach, Germany); and CD31-APC (clone WM59), CD105-PerCP/Cy5.5 (clone 43A3), and CD146-APC/Fire750 (clone P1H12) from BioLegend (San Diego, CA, USA). Antibodies were diluted according to the manufacturer’s instructions. Cells were incubated for 30 min at 4 °C in the dark, washed once with FACS buffer, and immediately analyzed. At least 30,000 events per sample were acquired using a CytoFlex flow cytometer (Beckman Coulter, Fullerton, CA, USA). Analysis was conducted with CytExpert software (Beckman Coulter).

### MSC secretome production and EV isolation

MSCs at 90% confluence were washed twice with PBS to remove residual growth media, then detached and reseeded at a density of 1 × 10^6^ cells/mL in 24-well plates (0.5 mL per well) using serum-free DMEM/F12. After four days of conditioning, cell viability was checked with a NucleoCounter NC-3000 (Chemometec, Allerod, Denmark). The culture supernatants (secretomes) were collected, centrifuged at 300 × *g* for 10 min at room temperature (RT) to remove cellular debris, and filtered through a 0.22 μm filter. Clarified secretomes were aliquoted and stored at -80 °C until further analyses. To collect EVs, secretomes were diluted 1:9 in PBS (1 mL secretome, corresponding to 1 × 10^6^ secreting cells) to a final volume of 10 mL and subjected to ultracentrifugation at 100,000 × *g* for 9 h at 4 °C using an Optima L-90K Ultracentrifuge (Beckman Coulter) equipped with a Type 70.1 Ti Fixed-Angle Titanium Rotor. After a PBS wash in the same conditions, pellets were either directly processed or suspended in an appropriate volume of PBS depending on the analysis.

### ELISA characterization of MSC secretomes

Secretomes were analyzed using the Quantibody® Human Cytokine Array 4000 Kit (RayBiotech, Peachtree Corners, GA, USA), following the manufacturer’s instructions. Samples were diluted 1:1 prior to analysis. Only cytokines consistently detected above the assay’s lower limit of detection across all 12 samples, or consistently undetectable in all replicates (*n* = 3) of one or more specific conditions but present in all remaining samples, were included in the final analysis. Concentrations were adjusted for the dilution factor and are reported as pg / mL (equivalent to pg/10^6^ cells).

### Protein-protein interaction networks

Protein-protein interaction (PPI) networks were constructed using the STRING database (v12.0; http://www.string-db.org)^[22]^ based on the set of proteins identified by enzyme-linked immunosorbent assay (ELISA). The following parameters were applied: (i) organism: Homo sapiens; (ii) network edge meaning: evidence; (iii) active interaction sources: experimental data and curated databases; and (iv) minimum required interaction score: medium confidence (0.400).

### Transmission electron microscopy analysis (TEM) of MSC-EVs

A 10 µL aliquot of each sample was deposited onto formvar carbon-coated copper grids and allowed to adsorb at RT for 10 min. Excess liquid was carefully removed by blotting with filter paper. Negative staining was then performed by incubating the grids with 2% (w/v) aqueous uranyl acetate for 10 min. After staining, excess reagent was blotted off, and the grids were air-dried at RT. Imaging was carried out using a TALOS L120C transmission electron microscope (Thermo Fisher Scientific) operated at an acceleration voltage of 120 kV.

### Nanoparticle tracking analysis (NTA) of MSC-EVs

Samples were diluted in PBS and analyzed using the NanoSight NS-300 system (NanoSight Ltd., Amesbury, UK). For each sample, five 60 s videos were recorded under controlled temperature conditions. EV size distribution and concentration were determined using Nanoparticle tracking analysis (NTA) software version 3.4, which provides high-resolution particle tracking and quantitative analysis.

### Flow cytometry characterization of MSC-EVs

Samples were either left unstained, stained with 10 μM carboxyfluorescein succinimidyl ester (CFSE) for 1 h at 37 °C in the dark, or double-stained with CFSE followed by incubation with allophycocyanin (APC)-conjugated antibodies for 30 min at 4 °C. Antibodies (BioLegend) were tested individually and included: anti-CD9 (clone H19a), CD63 (clone H5C6), CD73 (clone AD2), CD81 (clone 5A6), and CD90 (clone 5E10). Following staining, samples were further diluted and analyzed on a CytoFLEX flow cytometer (Beckman Coulter). Instrument calibration and nanometric gate settings were performed using a mixture of fluorescein isothiocyanate (FITC)-fluorescent nanobeads (100, 160, 200, 240, 300, 500, and 900 nm; Biocytex, Marseille, France). A minimum of 10,000 events was acquired per sample.

### Identification of miRNAs associated with MSC-EVs

To assess procedural efficiency and enable quantification, a synthetic spike-in (ath-miR-159) was included in each sample before RNA extraction. RNA extraction, complementary DNA (cDNA) synthesis and quantitative reverse transcription-polymerase chain reaction (qRT-PCR) analysis were performed as previously described^[23]^. Only miRNAs detected above the assay’s lower limit of detection across all 12 samples, or consistently undetectable in all replicates (*n* = 3) of one or more specific conditions but present in all remaining samples, were included in the final analysis. Data normalization across samples was carried out using the global mean method^[24]^ and miRNA values are reported as normalized C_RT_ (relative threshold cycle) values.

### Functional enrichment analysis of MSC-EV miRNAs

Functional enrichment analysis of MSC-EV miRNAs was performed using the miRNA Enrichment Analysis and Annotation Tool (miEAA, https://ccb-compute2.cs.uni-saarland.de/mieaa/)^[25]^, a web-based platform designed for comprehensive miRNA set analysis. Gene Ontology (GO) annotations were evaluated. P values were adjusted for multiple testing using the Benjamini-Hochberg false discovery rate (FDR) method, with adjustments applied independently for each category. Enrichment results with adjusted P values (FDR) below 0.05 were considered statistically significant. The minimum required number of hits per subcategory was two.

### Computational analyses

Principal component analysis (PCA) and hierarchical clustering were performed using the ClustVis web tool (https://biit.cs.ut.ee/clustvis/)^[26]^. Data transformation included natural logarithm ln(x + 1) when factor concentrations in pg/ml were near zero. No transformation was applied for C_RT_ polymerase chain reaction (PCR) values. No row centering or unit variance scaling was applied. PCA was conducted using singular value decomposition with imputation for missing values.

### Effect of secretomes on chondrocyte proliferation

Human immortalized healthy chondrocytes (INS-CI-1006; InSCREENeX, Braunschweig, Germany) at passage 11 were cultured in DMEM/F12 supplemented with 10% FBS. Cells were seeded at 10,000 cells/cm^2^ in 96-well plates. After 8 h to allow attachment, the medium was replaced with 100 µL of one of the following: fresh complete medium, complete medium supplemented with 1 ng/mL IL-1β (Sino Biological, Eschborn, Germany), or 50 µL secretomes (corresponding to 5 × 10^4^ cells) 1-fold diluted in complete medium containing 1 ng/mL IL-1β (total volume of 100 µL). For wells with secretome dilutions, final concentrations of FBS remained at 10 %. All conditions were prepared in quadruplicate. Baseline cell numbers were assessed immediately in two wells per condition by removing supernatants and adding 90 µL fresh medium plus 10 µL Cell Counting Kit - 8 (CCK-8) solution (Sigma-Aldrich, Darmstadt, Germany). Plates were incubated at 37 °C and absorbance was measured at 450 nm using a microplate reader (VICTOR^TM^ X3, PerkinElmer, Waltham, Massachusetts, USA) at 15, 30, and 60 min. Background absorbance from cell-free wells (in duplicate) was subtracted. A calibration curve using 10,000, 20,000, 40,000, 60,000 and 80,000 cells/cm^2^ was generated to correlate absorbance with cell number. After 48 h, the remaining replicate wells were similarly assayed with CCK-8, and cell numbers were calculated from the calibration curve. Proliferation was determined by comparing cell numbers at 48 h relative to baseline.

### Effect of secretomes on chondrocyte inflammation

Immortalized chondrocytes were prepared as described above and seeded at 90,000 cells/cm^2^ in 24-well plates. Cells were treated with either fresh complete medium, fresh complete medium supplemented with 1 ng/mL IL-1β, or secretomes 1-fold diluted in fresh complete medium containing 1 ng/mL IL-1β. For wells receiving diluted secretomes, the final concentrations of FBS were maintained at 10%. After 48 h, supernatants were removed, and total RNA was extracted using the RNeasy® Mini Kit (Qiagen) according to the manufacturer’s protocol. cDNA synthesis was performed using the iScript^TM^ cDNA Synthesis Kit (Bio-Rad Laboratories Srl, Segrate, Italy). Gene expression analysis of *Chatepsin S* (*CTSS*), *Interleukin 1B* (*IL-1β*), *IL-6*, *IL-8*, *C-C Motif Chemokine Ligand 5* (*CCL5*) and *indoleamine 2,3 dioxygenase 1* (*IDO1*) was conducted using iTaq Universal SYBR Green Supermix (Bio-Rad Laboratories Srl) on a CFX Opus Real-Time PCR System (Bio-Rad Laboratories Srl). TATA-Box Binding Protein for TBP (*TBP*) and Ribosomal Protein Lateral Stalk Subunit P0 for RPLP0 (*RPLP0*) were used as reference genes. Sequences will be provided upon request.

### Statistical analyses

Data are presented as mean ± standard deviation (SD) unless otherwise stated. Data visualization was performed using violin plots with truncated tails and Tukey whiskers. Normality of data distribution was assessed by the Shapiro-Wilk test (α = 0.05). With normal data, comparative analyses were conducted using one-way analysis of variance (ANOVA). With non-normal data, comparative analyses were conducted using the Kruskal-Wallis non-parametric test. Results are based on at least three independent experiments. Statistical analyses were performed using GraphPad Prism 8 (GraphPad Software, La Jolla, CA, USA), with significance defined as *P* ≤ 0.05. Values meeting this criterion were considered statistically significant.

## RESULTS

### MSCs immunophenotype

**Figure 1:**
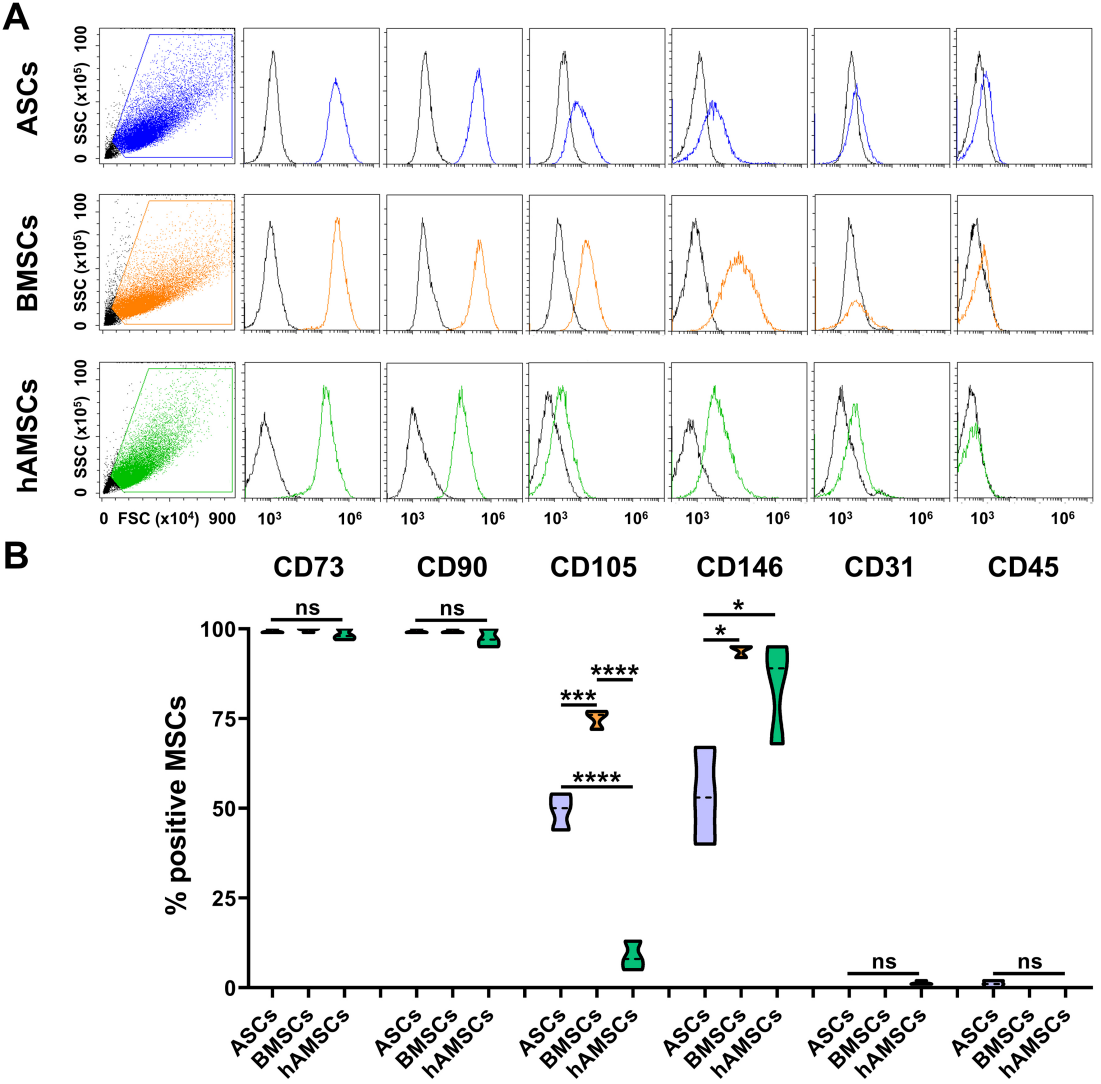
MSC immunophenotype. (A) Cytograms of markers tested for each MSC type of the study. A representative donor is shown; (B) Percentage of positive cells for both MSCs (CD73/90/105/146) and hemato-endothelial (CD31/45) markers (*N* = 3; ^*^P ≤ 0.05, ^***^P ≤ 0.001, ^****^P ≤ 0.0001). After assessment of normality of data distribution by the Shapiro-Wilk test, statistical significance was determined using one-way ANOVA. Data presented in PowerPoint. MSC: Mesenchymal stromal cell; ASCs: adipose-derived mesenchymal stromal cells; BMSCs: bone marrow-derived mesenchymal stromal cells; hAMSCs: human amniotic mesenchymal stromal cells; FSC: forward scatter; SSC: side scatter; CD: cluster of differentiation; ANOVA: analysis of variance; ns: not significant.

All MSC populations were strongly positive for the canonical lineage markers CD73 and CD90 [Figure 1A and B]. Expression of CD105 was significantly higher in BMSCs (75% ± 2%) compared to ASCs (45% ± 4%), and was minimally expressed in hAMSCs (9% ± 3%). CD146 was highly expressed in both BMSCs (94% ± 1%) and hAMSCs (84% ± 12%), while lower levels were observed in ASCs (53% ± 11%). All MSC types were negative for the hematopoietic and endothelial markers CD31 and CD45, respectively, confirming the absence of contaminating cell populations.

### MSCs secreted factors

**Figure 2:**
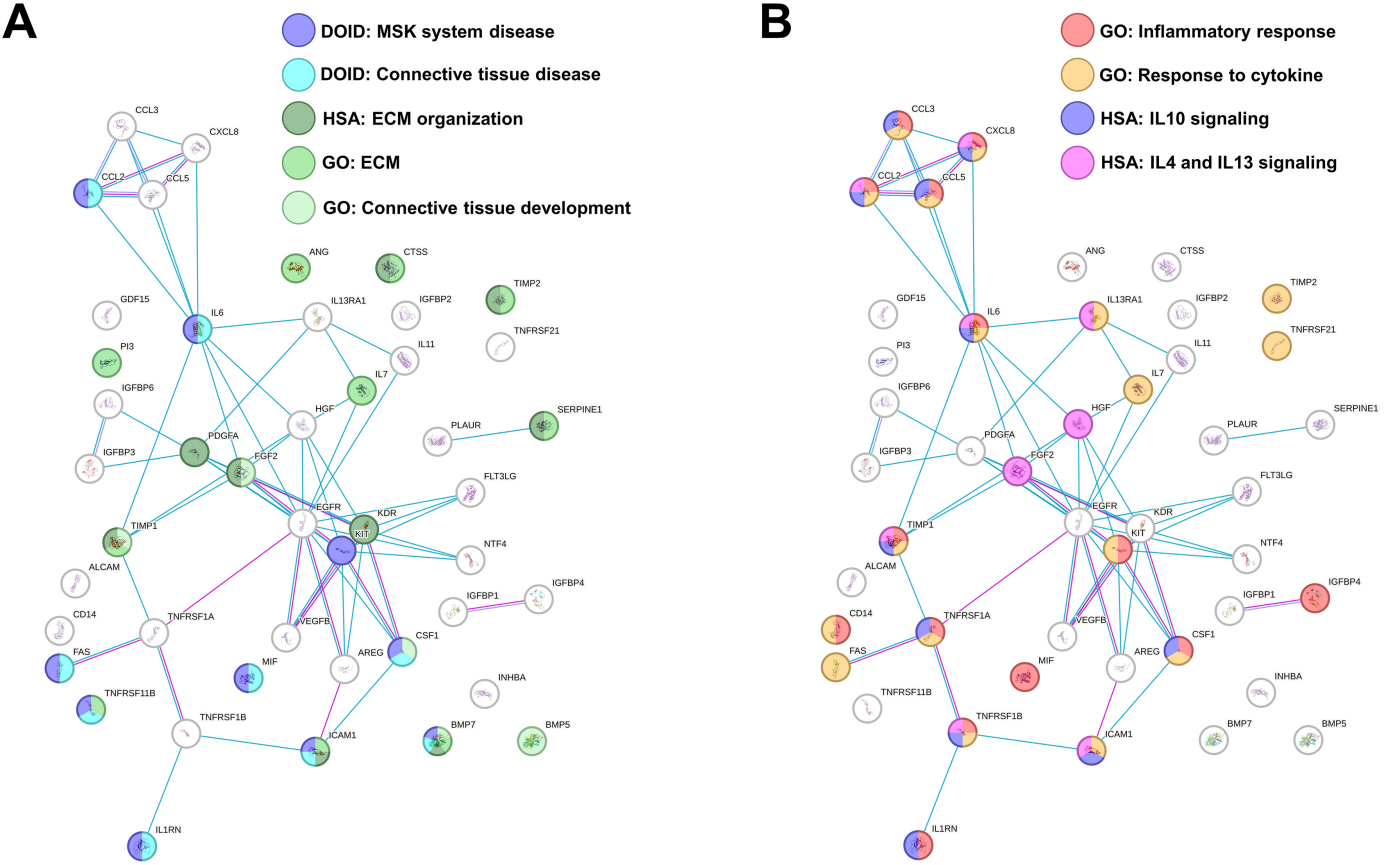
Functional association network for identified secreted factors. (A) Protein-protein interaction levels for proteins shared in MSC secretome, mined using STRING, and related to MSK and ECM terms. Blue connections: Proteins with known interactions based on curated databases; violet connections: proteins with experimentally determined interactions; Empty nodes: proteins of unknown 3D structure; filled nodes: known or predicted 3D structure; (B) Protein-protein interaction networks for proteins shared in MSC secretome, mined using STRING, and related to inflammation terms. For the color code, refer to (A). Data presented in PowerPoint. MSC: Mesenchymal stromal cell; MSK: musculoskeletal; ECM: extracellular matrix; GO: Gene Ontology; DOID: Disease Ontology ID; HSA: Homo sapiens pathway (Reactome); IL: interleukin; STRING: Search Tool for the Retrieval of Interacting Genes/Proteins.

At the time of secretome collection, cell viability after starvation was ≥ 90% in all preparations. Out of the 200 analyzed factors, 45 were identified as either commonly present across all secretomes or uniquely present/absent in one or two MSC sources compared to the others [Table 1 and Supplementary Table 1]. IL7 and Neurotrophin 4 (NTF4) were ASC-specific, whereas Interleukin 13 Receptor Subunit Alpha 1 (IL13RA1) was distinctive of BMSCs and hAMSCs. Functional enrichment analysis identified ten factors associated with musculoskeletal system disease (DOID:17), particularly with connective tissue disorders (DOID:65) [Table 2]. In this frame, nine factors were related to the Reactome pathway extracellular matrix (ECM) organization (HSA-1474244; Table 2) and 10 to the GO term ECM (GO:0031012; Table 2). Intriguingly, five molecules belong to the GO term Connective tissue development (GO:0061448, Table 2), reinforcing the notion of a direct impact on cartilage and its structure. Connected to the concept of tissue homeostasis regulation and related to the OA phenotype, 14 factors also shaped the GO term “Inflammatory response” (GO:0006954; Table 2), which overlaps with molecules in the GO term “Response to cytokine” (GO:0034097; Table 2). The majority of these proteins belonged to two GO terms related to anti-inflammatory Reactome pathways such as Interleukin-10 signaling (HSA-6783738; Table 2) and Interleukin-4 and Interleukin-13 signaling (HSA-6785807; Table 2). In this path, IL-6 and IL-1 receptor antagonist gene (IL1RN) were part of the GO term Negative regulation of Interleukin-1-mediated signaling pathway (GO:2000660). An overall functional picture of MSC factors is present in Figure 2A and B, showing how ECM and inflammation-modulating players are only partially overlapping.

**Table 1:** Factors detected in MSC secretomes

	**pg/mL**			**RATIO**		
	**ASCs**	**BMSCs**	**hAMSCs**	**ASCs *vs.* BMSCs**	**ASCs *vs.* hAMSCs**	**BMSCs *vs.* hAMSCs**
**ALCAM**	1,517	191	386	7.9^**^	3.9^**^	
**ANG**	70	280	199			
**AREG**	566	462	379			
**BMP5**	1,783	3,159	3,189			
**BMP7**	5,994	3,274	3,759			
**CCL2**	825	504	496			
**CCL3**	88	573	255			
**CCL5**	1,060	185	150	5.7^***^	7.1^***^	
**CD14**	556	2,423	3,140			
**CSF1**	350	188	156		2.2^**^	
**CTSS**	214	491	443			
**CXCL8**	606	420	492			
**EGFR**	1,752	961	878			
**FAS**	267	66	52	4.1^***^	5.2^***^	
**FGF2**	1,001	733	751			
**FLT3LG**	23	6	5	4.0^**^	4.9^**^	
**GDF15**	1,089	1,115	1,126			
**HGF**	174	717	720	0.2^****^	0.2^****^	
**ICAM1**	10,974	4,937	5,600			
**IGFBP1**	2,123	400	8,976			
**IGFBP2**	277	541	4,983			
**IGFBP3**	9,796	10,058	10,431			
**IGFBP4**	16,509	127,248	110,389			
**IGFBP6**	6,912	9,682	6,259			
**IL-11**	5,040	34,130	26,076	0.1^*^		
**IL13RA1**	ND	172	116	L	L	
**IL1RN**	299	239	179			
**IL-6**	14,371	12,372	16,383			
**IL7**	150	ND	ND	G	G	
**INHBA**	8,990	8,829	5,755			
**KDR**	117	204	215			
**KIT**	13	15	19			
**MIF**	6,689	6,512	7,960			
**NTF4**	48	ND	ND	G	G	
**PDGFA**	20	45	135			
**PI3**	2,316	1,907	1,697			
**PLAUR**	4,229	8,442	6,067			
**SERPINE1**	7,871	6,577	6,535			
**TIMP1**	11,208	10,937	8,640			
**TIMP2**	11,501	11,524	6,306			
**TNFRSF11B**	447	284	350			
**TNFRSF1A**	3,001	3,836	3,442			
**TNFRSF21**	28	48	41			
**TNFSRSF1B**	729	1,650	1,184			
**VEGF**	23,965	20,395	22,583			

^*^P ≤ 0.05; ^**^P ≤ 0.01; ^***^P ≤ 0.001 and ^****^P ≤ 0.0001. Ratio values not reaching a statistical significance (ns) are not shown. Mean of 3 donors per MSC type. After assessment of normality of data distribution by the Shapiro-Wilk test, statistical significance was determined using one-way ANOVA. MSC: Mesenchymal stromal cell; ASCs: adipose-derived mesenchymal stem cells; BMSCs: bone marrow-derived mesenchymal stem cells; hAMSCs: human amniotic mesenchymal stem cells; pg/mL: picograms per milliliter; ALCAM: activated leukocyte cell adhesion molecule; ANG: angiogenin; AREG: amphiregulin; BMP: bone morphogenetic protein; CCL: C-C motif chemokine ligand; CD14: cluster of differentiation 14; CSF1: colony-stimulating factor 1; CTSS: cathepsin S; CXCL8: C-X-C motif chemokine ligand 8; EGFR: epidermal growth factor receptor; FAS: Fas cell surface death receptor; FGF2: fibroblast growth factor 2; FLT3LG: Fms-related tyrosine kinase 3 ligand; GDF15: growth differentiation factor 15; HGF: hepatocyte growth factor; ICAM1: intercellular adhesion molecule 1; IGFBP: insulin-like growth factor binding protein; IL: interleukin; IL13RA1: interleukin-13 receptor subunit alpha 1; IL1RN: interleukin-1 receptor antagonist; INHBA: inhibin subunit beta A; KDR: kinase insert domain receptor; KIT: KIT proto-oncogene receptor tyrosine kinase; MIF: macrophage migration inhibitory factor; NTF4: neurotrophin 4; PDGFA: platelet-derived growth factor subunit A; PI3: peptidase inhibitor 3; PLAUR: plasminogen activator, urokinase receptor; SERPINE1: serpin family E member 1; TIMP: tissue inhibitor of metalloproteinases; TNFRSF11B: tumor necrosis factor receptor superfamily member 11B; TNFRSF1A: tumor necrosis factor receptor superfamily member 1A; TNFRSF21: tumor necrosis factor receptor superfamily member 21; TNFRSF1B: tumor necrosis factor receptor superfamily member 1B; VEGF: vascular endothelial growth factor; ANOVA: analysis of variance; ND: not detected; ns: not significant; L: lost; G: gained.

**Table 2:** Functional enrichment of MSCs factors

		**Factors**	**FDR**
**Disease**	DOID17: MSK disease	BMP7, CCL2, CSF1, FAS, KIT, ICAM1, IL1RN, IL-6, MIF, TNFRSF11B	4.94E-02
	DOID65: Connective tissue disease	BMP7, CCL2, CSF1, FAS, ICAM1, IL1RN, IL-6, MIF, TNFRSF11B	1.87E-02
**Gene ontology term**	HSA-1474244: ECM organization	BMP7, CTSS, FGF2, ICAM1, KDR, PDGFA, SERPINE1, TIMP1, TIMP2	5.62E-06
	GO:0031012: Extracellular matrix	ANG, BMP7, CTSS, ICAM1, IL7, PI3, SERPINE1, TIMP1, TIMP2, TNFRSF11B	1.70E-04
	GO:0061448: Connective development	BMP5, BMP7, CSF1, FGF2, TIMP1	6.90E-03
	GO:0006954: Inflammatory response	CCL2, CCL3, CCL5, CD14, CFS1, CXCL8, IGFBP4, IL1RN, IL-6, KIT, MIF, TIMP1, TNFRSF1A, TNFRSF1B	2.48E-09
	GO:0034097: Response to cytokine	CCL2, CCL3, CCL5, CD14, CSF1, CXCL8, FAS, ICAM1, IL-6, IL13RA1, IL17, KIT, TIMP1, TIMP2, TNFRSF1A, TNFRSF1B, TNFRSF21	2.87E-10
	HSA-67883783: IL-10 signaling	CCL2, CCL3, CCL5, CSF1, CXCL8, ICAM1, IL1RN, IL-6, TIMP1, TNFRSF1A, TNFRSF1B	7.24E-16
	HSA-6785807: IL-4/IL-13 signaling	CCL2, CXCL8, FGF2, HGF, ICAM1, IL-6, IL13RA1, TIMP1, TNFRSF1B	2.06E-09

MSCs: Mesenchymal stem cells; DOID: Disease Ontology ID; MSK: musculoskeletal; BMP: bone morphogenetic protein; CCL: C-C motif chemokine ligand; CSF1: colony stimulating factor 1; FAS: Fas cell surface death receptor; KIT: KIT proto-oncogene receptor tyrosine kinase; ICAM1: intercellular adhesion molecule 1; IL1RN: interleukin 1 receptor antagonist; IL: interleukin; IL13RA1: interleukin 13 receptor subunit alpha 1; IL17: interleukin 17; MIF: macrophage migration inhibitory factor; TNFRSF: tumor necrosis factor receptor superfamily member; CTSS: Cathepsin S; FGF2: fibroblast growth factor 2; KDR: kinase insert domain receptor; PDGFA: platelet derived growth factor subunit A; SERPINE1: serpin family E member 1; TIMP: tissue inhibitor of metalloproteinases; ANG: angiogenin; PI3: peptidase inhibitor 3; CD14: cluster of differentiation 14; IGFBP4: insulin-like growth factor binding protein 4; HGF: hepatocyte growth factor; ECM: extracellular matrix; FDR: false discovery rate; HSA: Homo sapiens annotation; GO: Gene Ontology.

**Figure 3:**
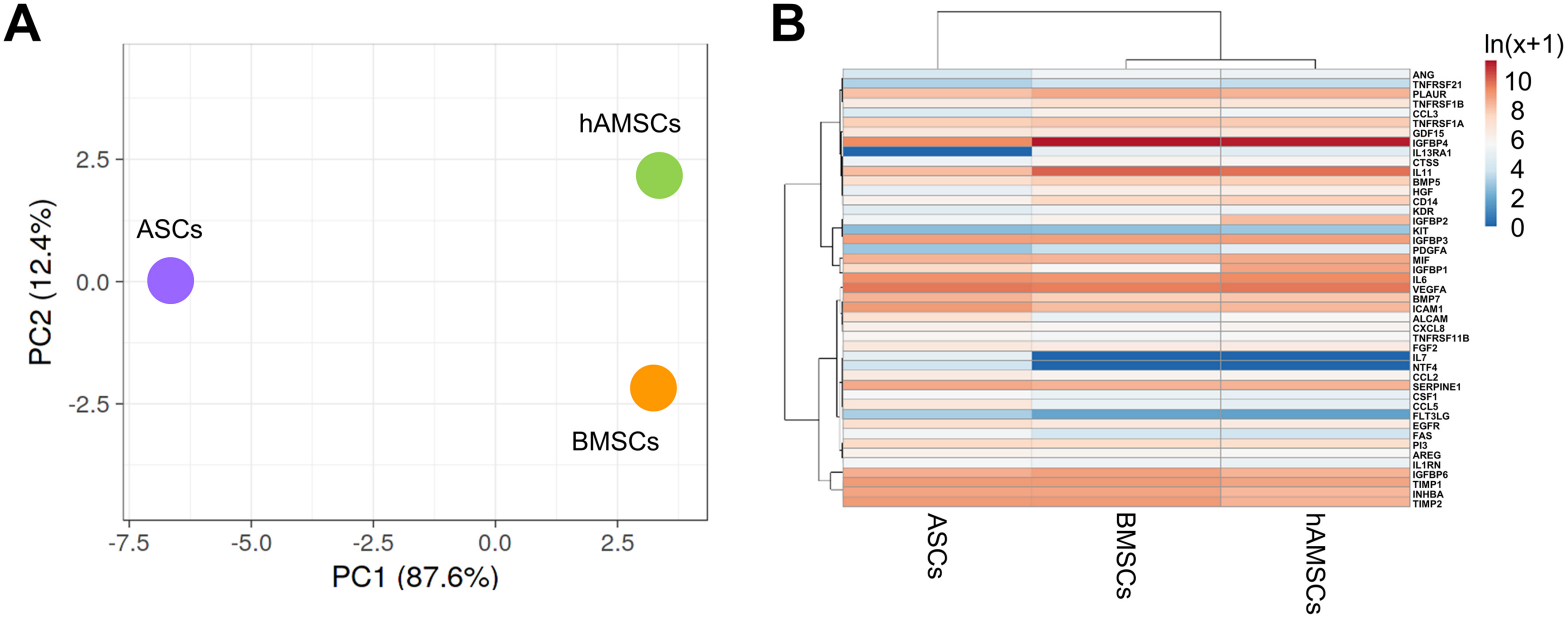
Secreted factor profile comparison between MSC types under study. (A) Principal component analysis of the ln(x+1)-transformed pg/ml MSC values of detected factors. The X and Y axes show Principal Component 1 and Principal Component 2, which explain 87.6% and 12.4% of the total variance, respectively; (B) Heat map of hierarchical clustering analysis of the ln(x+1)-transformed pg/mL MSCs values of detected factors with sample clustering tree at the top. Absolute expression levels reflect the color scale: red shades: high expression levels; blue shades: low expression levels. Data presented in PowerPoint. MSC: Mesenchymal stromal cell; hAMSCs: human amniotic mesenchymal stromal cells; BMSCs: bone marrow-derived mesenchymal stromal cells; ASCs: adipose-derived mesenchymal stromal cells; PCA: principal component analysis; PC1: principal component 1; PC2: principal component 2; ln: natural logarithm; VEGFA: vascular endothelial growth factor A; BMP7: bone morphogenetic protein 7; ALCAM: activated leukocyte cell adhesion molecule; IGFBP: insulin-like growth factor-binding protein; TIMP: tissue inhibitor of metalloproteinases; CTSS: cathepsin S; CSF1: colony-stimulating factor 1; NTF4: neurotrophin 4; SERPINE1: serpin family E member 1; AREG: amphiregulin.

We further compared the molecular signatures of the different secretomes. In a context of high similarity, BMSCs and hAMSCs resulted in the most correlated datasets (Spearman’s rank correlation coefficient, r = 0.939), while ASCs were the most distant (r = 0.885 *vs.* BMSCs and r = 0.893 *vs.* hAMSCs). This analysis was supported by PCA and clustering analyses [Figure 3A and B]. At the single-factor level, there were no proteins differentially released by BMSCs and hAMSCs, whereas ASCs showed four upregulated and two downregulated molecules. The most increased ones were Activated Leukocyte Cell Adhesion Molecule (ALCAM) (7.9-fold *vs.* BMSCs and 3.9 *vs.* hAMSCs), followed by CCL5 (5.7 and 7.1), Fas Cell Surface Death Receptor (FAS) (4.1 and 5.2) and Fms Related Receptor Tyrosine Kinase 3 Ligand (FLT3LG) (4.0 and 4.9). The reduced ones were Hepatocyte Growth Factor (HGF) (0.2 and 0.2) and IL-11 (0.1 *vs.* BMSCs and 0.2 *vs*. hAMSCs with a *P*-value of 0.1171). Functional analysis including also the MSC-type specific IL7, NTF4 and IL13RA1 did not allow the identification of Musculoskeletal or Connective tissue-diseases terms or OA-related biological processes, except the general GO term Negative regulation of apoptotic process (GO:0043066) framed by CCL5, FAS, HGF, IL7 and NTF4.

### MSC-EV characterization and immunophenotype

**Figure 4:**
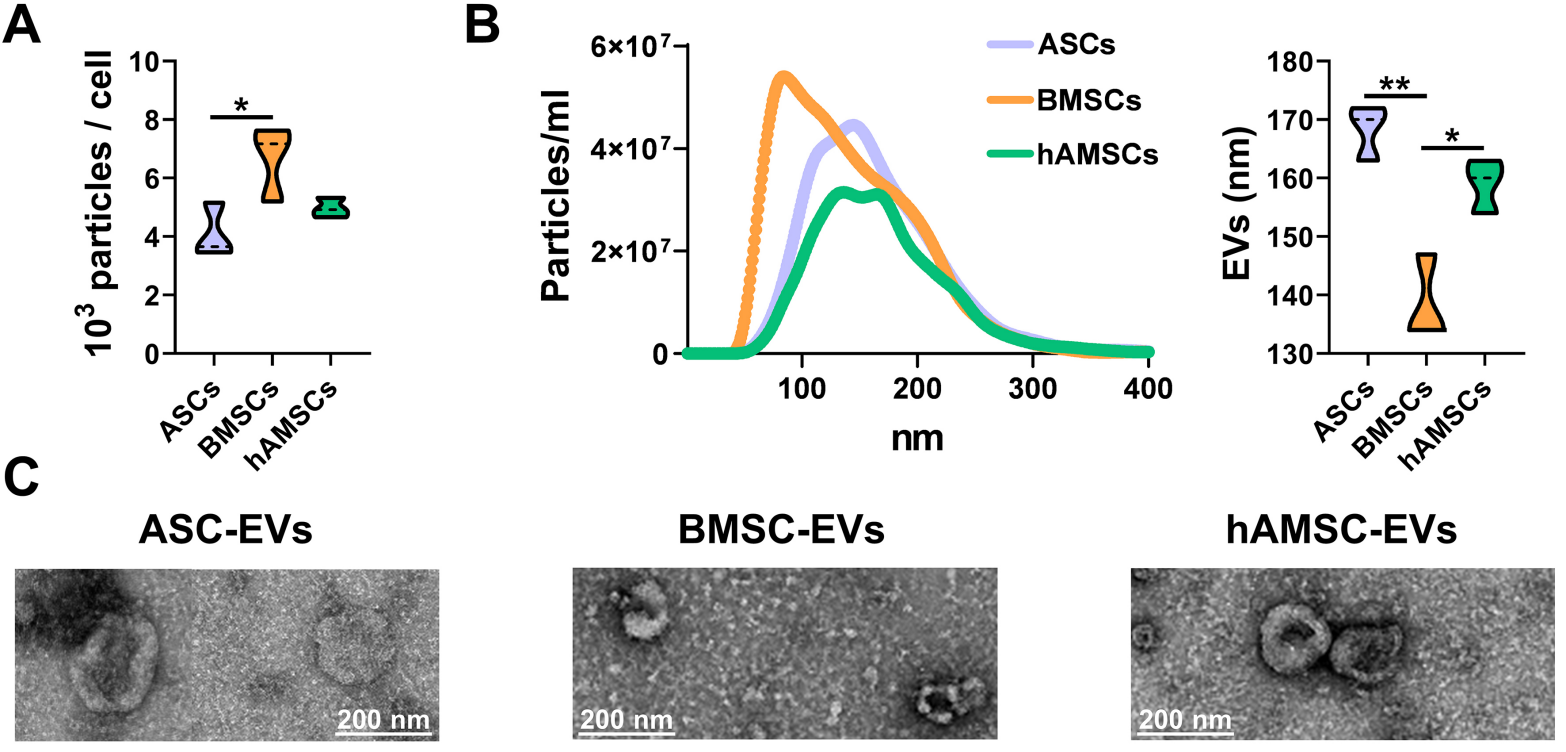
MSC-EV characterization. (A) Particles released per cell calculated from NTA data (*N* = 3; ^*^P ≤ 0.05). After assessment of normality of data distribution by the Shapiro-Wilk test, statistical significance was determined using ANOVA; (B) Particle size analysis using NTA (each curve was obtained by merging the data from three independent MSC-type donors). Mean size results are displayed as violin plots (*N* = 3; ^*^P ≤ 0.05, ^**^P ≤ 0.01). After assessment of normality of data distribution by the Shapiro-Wilk test, statistical significance was determined using one-way ANOVA; (C) Transmission electron microscopy images of particles released by ASC, BMSCs and hAMSCs. Scale bar is included in each micrograph. Data presented in PowerPoint. MSC: Mesenchymal stromal cell; ASC: adipose-derived mesenchymal stromal cell; BMSC: bone marrow-derived mesenchymal stromal cell; hAMSC: human amniotic mesenchymal stromal cell; EVs: extracellular vesicles; NTA: nanoparticle tracking analysis; TEM: transmission electron microscopy; ANOVA: analysis of variance; nm: nanometer.

**Figure 5:**
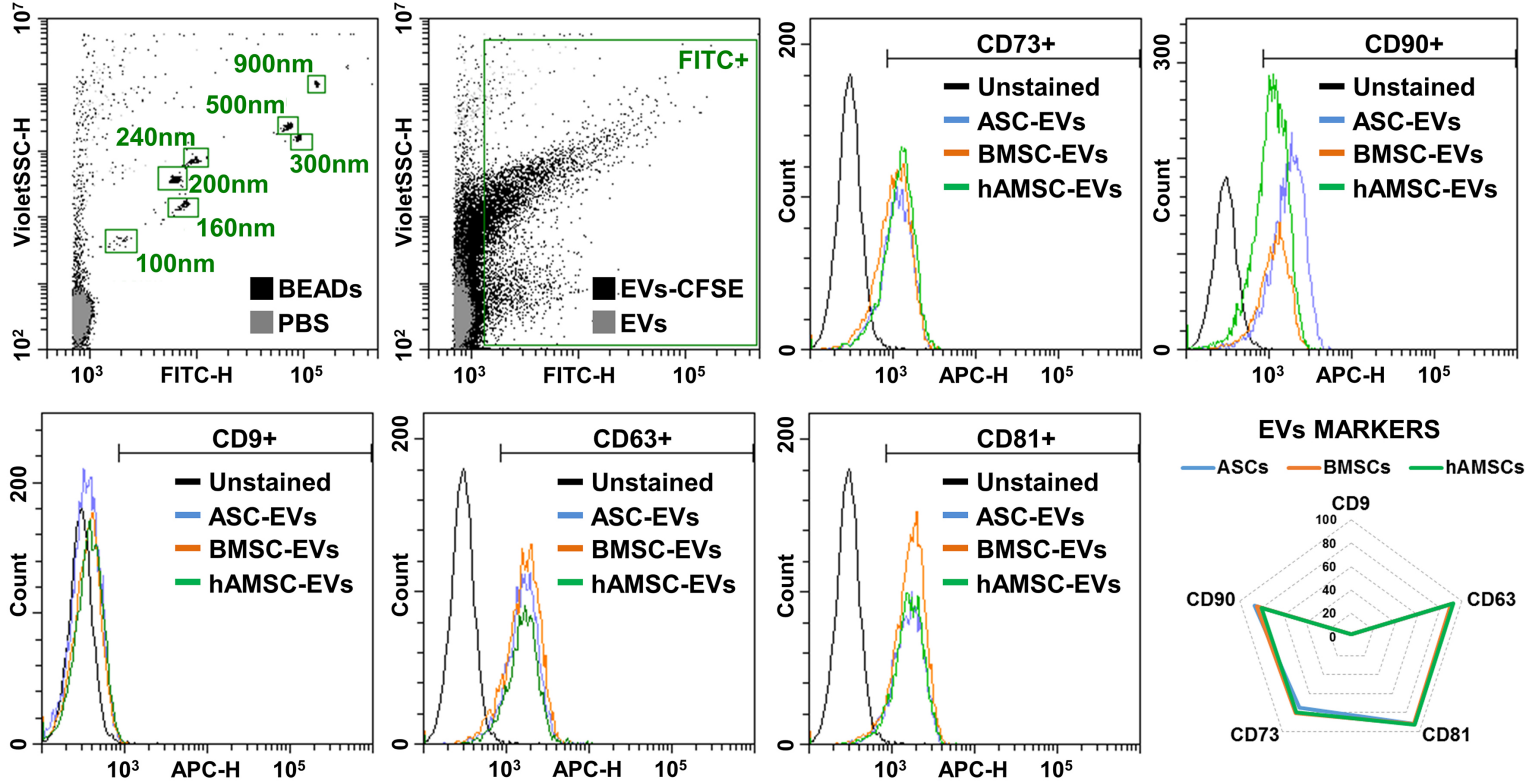
MSC-EV immunophenotype. Representative cytograms of EV (CD9/63/81) and MSC (CD73/90) markers tested in representative MSC-EVs after flow cytometer calibration with FITC-positive polystyrene beads of predetermined size (100, 160, 200, 240, 300, 500 and 900 nm) to confirm reliability of particle detection in the nanometric range. CFSE-stained only and antibody-unstained samples represent EVs from ASCs for clarity. No significant statistical difference (*P*-value ≤ 0.05, *N* = 3) emerged for tested markers. After assessment of normality of data distribution by the Shapiro-Wilk test, statistical significance was determined using one-way ANOVA. Data presented in PowerPoint. MSC: Mesenchymal stromal cell; ASC: adipose-derived mesenchymal stromal cell; BMSC: bone marrow-derived mesenchymal stromal cell; hAMSC: human amniotic mesenchymal stromal cell; EVs: extracellular vesicles; ASC-EVs: extracellular vesicles derived from ASCs; BMSC-EVs: extracellular vesicles derived from BMSCs; hAMSC-EVs: extracellular vesicles derived from hAMSCs; CD: cluster of differentiation; FITC: fluorescein isothiocyanate; APC: allophycocyanin; CFSE: carboxyfluorescein succinimidyl ester; PBS: phosphate-buffered saline; ANOVA: analysis of variance; SSC: side scatter; nm: nanometer.

BMSCs showed the highest release of nanoparticles (6.7 × 10^3^ ± 1.1 per cell in 96 h) compared with ASCs (4.1 × 10^3^ ± 0.8) and hAMSCs (5.0 × 10^3^ ± 0.3) [Figure 4A]. The nanoparticles from BMSCs were smaller (138 ± 6 nm) than those from the other sources (168 ± 4 nm for ASCs and 159 ± 4 nm for hAMSCs) [Figure 4B]. Isolated nanoparticles showed typical EV features by transmission electron microscopy (TEM) [Figure 4C]. Regardless of tissue source, EVs were positive for both vesicle markers CD63 and CD81 and MSC-lineage markers CD73 and CD90 [Figure 5]. CD9, another vesicle marker, was negative [Figure 5].

### MSC-EVs-associated miRNAs

**Figure 6:**
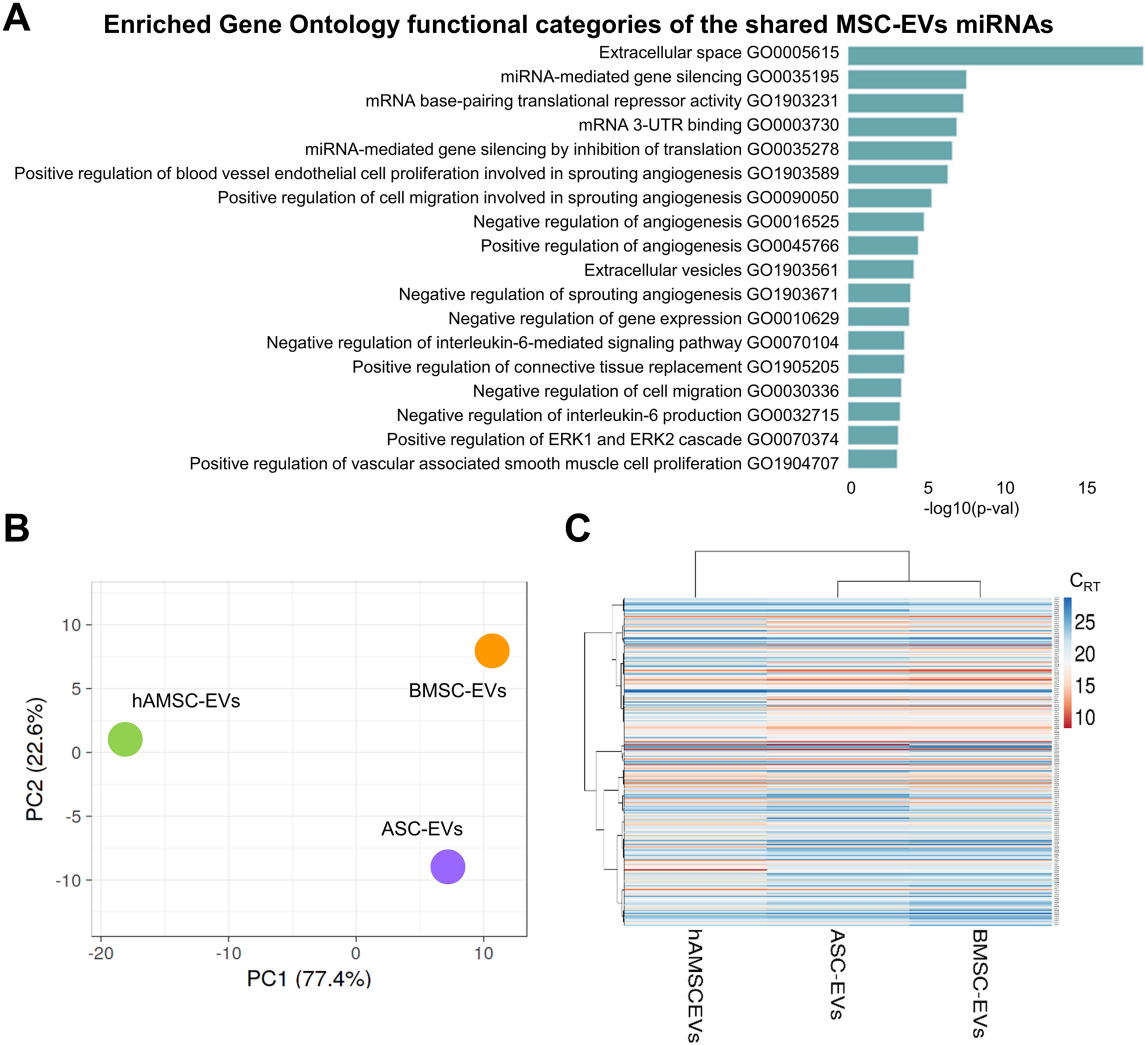
Comparison of EVs among MSC types under study. (A) Enriched GO functional categories of the shared MSC-EVs miRNAs. The vertical axis showed the GO category, and the horizontal axis showed the *P*-value (-log_10_); (B) Principal component analysis of the miRNA C_RT_ values. The X and Y axes show PC1 and PC2, which explain 77.4% and 22.6% of the total variance, respectively; (C) Heat map of hierarchical clustering analysis of miRNA C_RT_ values (mean of the three MSC-EVs samples for each type) with sample clustering tree at the top. Red shades: High expression levels; blue shades: low expression levels. Data presented in PowerPoint. MSC: Mesenchymal stromal cell; EVs: extracellular vesicles; ASC-EVs: extracellular vesicles derived from adipose-derived mesenchymal stromal cells; BMSC-EVs: extracellular vesicles derived from bone marrow-derived mesenchymal stromal cells; hAMSC-EVs: extracellular vesicles derived from human amniotic mesenchymal stromal cells; miRNA: microRNA; GO: Gene Ontology; PCA: principal component analysis; PC1: principal component 1; PC2: principal component 2; C_RT_: cycle relative threshold; ERK: extracellular signal-regulated kinase; UTR: untranslated region.

Out of the 784 analyzed miRNAs, 249 were identified as either commonly present across all secretomes or uniquely present/absent in one or two MSC sources compared to the others [Supplementary Table 2]. Overall, top enriched GO categories identified by EV-miRNAs were related to Extracellular space (GO:0005615) including Positive regulation of connective tissue replacement (GO:1905205), gene silencing (miRNA-mediated such as GO:0035195, GO:0003730 and GO:0035278), angiogenesis (both negative and positive, GO:0016525 and GO:0045766) based on endothelial cell proliferation and migration (GO:1903589 and GO:0090050) and IL-6 signaling and production (GO:0070104 and GO:0032715) [Figure 6A and Supplementary Table 3].

As for proteins, the molecular fingerprint of MSC-EVs was compared. In a context of high similarity, ASCs and BMSCs were the most correlated datasets (Spearman r = 0.968), while hAMSCs were the most distant (0.906 *vs*. ASCs and 0.898 *vs*. BMSCs). This analysis was supported by PCA and clustering analyses [Figure 6B and C]. To highlight the biological significance of the identified miRNAs, only those in the first quartile of expression in each MSC-EV type were selected, for a total of 78 candidates [Supplementary Table 4], with 38 showing differential expression [Supplementary Table 4]. The most upregulated miRNA was miR-146a-5p, which showed almost a 1,000-fold increase in hAMSC-EVs compared with the other MSC-EVs. Consistent with correlation analyses, ASC-EVs and BMSC-EVs shared a conserved modulation pattern versus hAMSC-EVs for several miRNAs, including miR-138-5p (96- and 219-fold upregulation, respectively), miR-137-3p (123- and 39-fold, respectively), let-7a-5p/7e-5p, and miR-145-5p/708-5p (4-7- and 14-15-fold, respectively). let-7b-5p, miR-30b-5p/30c-5p, and miR-140-5p showed a consistent 4- to 9-fold increase in EVs from adult sources. A few miRNAs were specific to ASC-EVs compared with hAMSC-EVs, including miR-31-5p/3p (9-fold). Several other miRNAs showed weaker modulation, ranging from 2- to 4-fold in both directions.

Eventually, the most abundant EV-miRNAs were compared with those reported as involved in OA pathology or therapy^[27,28]^ [Table 3]. Sixteen miRNAs with protective functions and 10 with pathogenic roles were identified. Additionally, six miRNAs with overlapping activity were found. To assign an overall functional direction for miRNAs in MSC-EVs, their genetic weight within the first quartile group was calculated across all datasets [Table 3]. In all MSC types, protective signals greatly outweighed destructive ones, with BMSC-EVs performing best in both the amount of positive miRNAs (39% of the genetic weight *vs*. 32% for ASC-EVs and hAMSC-EVs) and the prot/dest ratio (3.4 *vs*. 2.1 and 1.5, respectively). This result can be largely attributed to the presence of four miRNAs with therapeutic activity (miR-125b-5p, miR-24-3p, miR-222-3p, miR-193b-3p) and only one highly expressed pathogenic miRNA (miR-21-5p), followed by miR-19b-3p at considerably lower levels. Notably, the highly abundant and hAMSC-EV-specific miR-146a-5p fell into the group of molecules with dual function, along with the adult MSC-defining miR-145-5p.

**Table 3:** OA-related MSC-EV miRNAs

**Protective** **(I° quartile genetic weight %)**				**Overlapping** **(I° quartile genetic weight %)**				**Destructive** **(I° quartile genetic weight %)**			
	ASCs	BMSCs	hAMSCs		ASCs	BMSCs	hAMSCs		ASCs	BMSCs	hAMSCs
miR-17-5p	0.3	0.6	0.7	miR-26a-5p	1.0	1.0	0.6	miR-16-5p	0.2	0.2	0.1
miR-24-3p	9.8	13.4	6.2	miR-27b-3p	0.1	0.2	0.1	miR-19b-3p	1.4	1.3	2.9
miR-26b-5p	0.1	0.2	0.3	miR-140-5p	0.1	0.1	0.01	miR-21-5p	12.1	7.3	15.4
miR-27a-3p	0.2	0.2	0.1	miR-145-5p	3.3	5.9	0.4	miR-29c-3p	0.1	0.1	0.5
miR-29a-3p	0.5	0.8	0.4	miR-146a-5p	0.01	0.01	14.3	miR-30b-5p	0.5	1.0	0.2
miR-30a-5p	0.1	0.2	0.1	miR-221-3p	1.9	4.5	2.8	miR-34a-5p	0.7	0.8	0.4
miR-92a-3p	0.6	0.9	2.2					miR-138-5p	0.1	0.2	0.001
miR-125b-5p	12.9	6.0	11.9					miR-181a-5p	0.1	0.2	0.2
miR-130a-3p	0.2	0.4	0.4					miR-218-5p	0.1	0.1	0.2
miR-148a-3p	0.1	0.1	0.4					miR-483-5p	0.1	0.2	0.6
miR-152-3p	0.4	0.4	0.8								
miR-193b-3p	1.5	3.3	3.9								
miR-199a-3p	0.7	0.6	0.3								
miR-210-3p	1.0	1.0	1.2								
miR-222-3p	3.7	11.1	2.7								
miR-335-5p	0.1	0.2	0.2								
Total %	32	39	32		6	12	18		15	11	21
Prot/Des ratio	2.1	3.4	1.5								

OA: Osteoarthritis; MSC: mesenchymal stem cell; EV: extracellular vesicle; ASCs: adipose-derived stem cells; BMSCs: bone marrow-derived stem cells; hAMSCs: human amniotic mesenchymal stem cells; miRNA: microRNA; Prot/Des ratio: Protective-to-Destructive ratio.

### Effects of MSC secretomes on inflamed human chondrocytes

**Figure 7:**
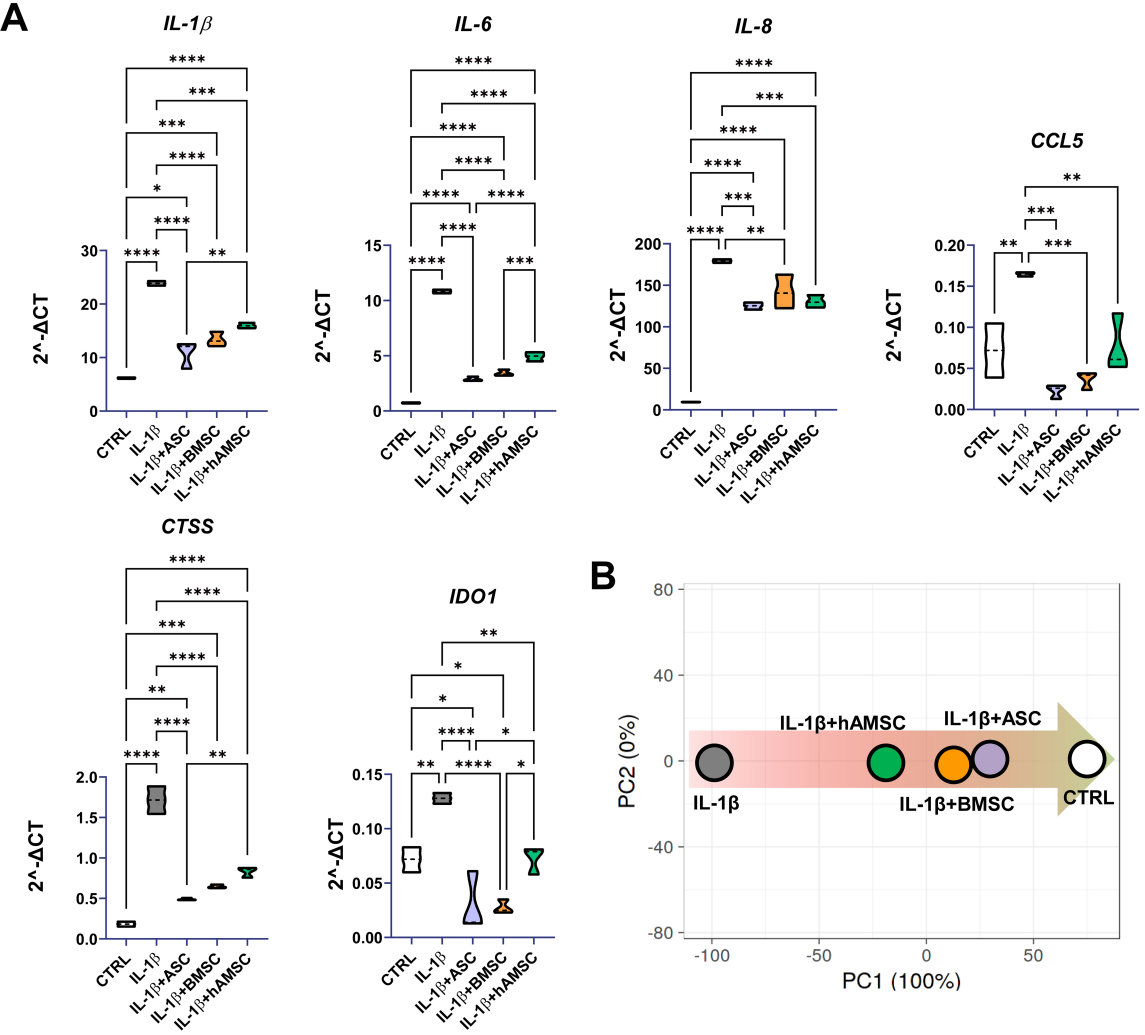
Effect of secretomes on inflamed chondrocytes. (A) Gene expression modulation (2^-ΔCt^) for chondrocytes exposed to IL-1β without and with secretomes at 1:1 dilution (^*^P ≤ 0.05, ^**^P ≤ 0.01, ^***^P ≤ 0.001, ^****^P ≤ 0.0001; *N* = 3). After assessment of normality of data distribution by the Shapiro-Wilk test, statistical significance was determined using one-way ANOVA; (B) Principal component analysis for 2^-ΔCt^ values of tested genes showing reversion of secretome-treated inflamed-chondrocytes signature towards healthy control samples. Data presented in PowerPoint. ASC: Adipose-derived mesenchymal stromal cell; BMSC: bone marrow-derived mesenchymal stromal cell; hAMSC: human amniotic mesenchymal stromal cell; CTRL: control; IL: interleukin; IL-1β: interleukin 1 beta; CCL5: C-C motif chemokine ligand 5; CTSS: cathepsin S; IDO1: indoleamine 2,3-dioxygenase 1; PCA: principal component analysis; PC1: principal component 1; PC2: principal component 2; ΔCt: delta cycle threshold; ANOVA: analysis of variance.

The effects of the MSC-derived secretomes on human chondrocytes were evaluated using an *in vitro* model of inflammation induced by IL-1β, a widely established approach for mimicking OA-like conditions and assessing the potential of novel therapeutic strategies. hAMSC-conditioned medium was the only one able to significantly reduce inflamed chondrocyte proliferation (CTRL 100% ± 7%, IL-1β 81% ± 2%, IL-1β + ASC 87% ± 13%, IL-1β + BMSC 84% ± 4%, IL-1β + hAMSC 57% ± 4%; IL-1β + hAMSC significantly different, *P*-value ≤ 0.05, *vs.* all other conditions; *n* = 3). Further, six genes involved in inflammation-dependent OA phenotype at different levels [*CTSS* for matrix remodeling; Interleukins (*IL-1/6/8*) as inflammatory cytokines; *CCL5* as inflammatory chemokine and *IDO1* as Wnt pathway activator and cartilage regeneration blocker] were tested [Figure 7A]. All secretomes were able to counteract inflammation-driven upregulation of the tested genes, although some differences emerged. ASC secretome was the best performer together with BMSC secretome, with no significant differences observed between the two. Although hAMSC secretome behaved in a similar fashion, reductions in *IL-1β*, *IL-6*, *CTSS* and *IDO1* were significantly lower compared with ASC-, BMSC- or both secretomes. Overall, the anti-inflammatory capacity in human chondrocytes was confirmed by PCA analysis, with ASCs and BMSCs again clustering together [Figure 7B].

## DISCUSSION

This study provides a comprehensive characterization of the secretomes derived from ASCs, BMSCs, and hAMSCs, with a particular focus on their relevance to OA pathophysiology. Through the integration of surface immunophenotyping, broad profiling of secretome factors and EV-associated miRNAs, and functional assessment in inflamed human chondrocytes, we identified both common mechanisms and source-specific molecular signatures potentially underlying their differential therapeutic efficacy.

All MSC populations fulfilled the minimal immunophenotypic criteria^[29]^, yet exhibited distinct expression patterns of regulatory surface markers with recognized roles in joint biology. BMSCs and ASCs showed the highest levels of CD105, a receptor involved in Transforming Growth Factor Beta (TGF-β) signaling^[30]^ and previously associated with cartilage repair capacity and joint homing^[31]^. Consistently, CD105^+^ MSCs have been reported to display stronger chondrogenic potential than their CD105^-^ counterparts^[32,33]^. The nearly absent CD105 expression observed in hAMSCs may therefore indicate a reduced responsiveness to TGF-β-mediated remodeling, potentially affecting their therapeutic contribution in osteoarthritic conditions. Regarding CD146, all MSC types expressed this marker, with ASCs displaying the lowest levels, although flow cytometry plots revealed a homogeneous expression across the cell population. The presence of CD146 is relevant to joint preservation, as CD146^+^ MSCs have been shown to be more active in cartilage regeneration than CD146^-^ MSCs^[34,35]^. Collectively, these findings indicate that all MSC sources investigated possess features conducive to promoting joint homeostasis, although subtle phenotypic differences may underlie source-specific functional outcomes.

In this context, factor profiling revealed that all MSC secretomes were enriched in proteins involved in ECM organization and connective tissue development, consistent with their potential to modulate cartilage integrity. Among the 200 factors analyzed, 45 were uniquely distributed or shared among specific MSC subsets. Notably, enrichment analysis identified 10 molecules associated with musculoskeletal and connective tissue disorders, as well as with pathways regulating ECM dynamics. These included several ECM-modifying enzymes and regulators implicated in osteoarthritic cartilage homeostasis. For instance, the highly abundant Tissue Inhibitor Of Metalloproteinase 1 and 2 (TIMP1 and TIMP2) are key inhibitors of matrix metalloproteinases (MMPs), thereby preventing ECM degradation^[36]^. Similarly, the prevalent SerpinE1 protects against cartilage collagen breakdown by inhibiting proteolytic activators of MMPs^[37]^. Consistently, Bone Morphogenetic Protein 7 (BMP7) has been reported to counteract the fibrocartilaginous chondrocyte phenotype by suppressing fibrotic type I collagen expression^[38]^. Furthermore, 14 proteins were associated with inflammatory response pathways, highlighting the dual structural and immunomodulatory roles of MSC secretomes. Of particular relevance to OA, the secretomes showed marked enrichment in anti-inflammatory cascades, notably those mediated by IL-10^[39]^ and IL-4/IL-13^[40]^, both known to mitigate the IL-1-driven catabolic environment characteristic of osteoarthritic cartilage^[41]^. The presence of IL1RN, a natural antagonist of the IL-1β/Nuclear Factor Kappa B (NF-κB) signaling axis^[42]^, further underscores the potential of MSC secretomes to attenuate inflammatory stimuli that drive chondrocyte catabolism and matrix degradation in OA. Despite these shared features, source-dependent differences emerged. ASCs exhibited significantly distinct levels of molecules involved in immune cell communication and tissue regeneration, suggesting a broader modulatory range. For example, ASCs upregulated factors implicated in cartilage turnover and chondrocyte-matrix interactions, such as ALCAM^[43]^ and CCL5^[44]^, while downregulating HGF^[45]^ and IL-11^[46]^. The opposing expression patterns of these molecules compared with other MSC types make it challenging to predict the net functional outcome of ASC secretomes. To provide some perspective, IL-11, the most abundant differentially expressed protein, is associated with OA pathology and may contribute to disease progression^[47]^; thus, its reduced expression in ASCs could potentially shift the secretome’s balance toward a more protective profile. However, previous studies have also reported a protective role of IL-11 in human articular cartilage^[48]^, indicating that its function in OA remains context-dependent. For these reasons, the overall biological activity of the MSC secretomes should be interpreted as the integrated outcome of all contributing molecular components rather than single-factor effects.

EV production and molecular fingerprinting further underscored intrinsic differences among MSC sources. BMSCs released the highest number of nanoparticles, potentially reflecting enhanced paracrine activity. However, their smaller EV size suggests that, despite quantitative disparities, the total vesicular volume and density of transported signals may be comparable across MSC types. Overall, the 249 miRNAs shared among all EV samples were associated with biological processes highly relevant to OA, including ECM remodeling, angiogenesis, cytokine response, and apoptotic regulation. Enrichment in the GO terms “positive regulation of connective tissue replacement” and “IL-6 signaling” highlights the potential of these vesicles to concomitantly modulate structural repair and immune response within the osteoarthritic joint. Despite these shared features, the EV miRNA cargo composition differed across MSC sources, a critical determinant of their regulatory function alongside soluble factor modulation. ASC- and BMSC-derived EVs displayed a higher degree of correlation, whereas hAMSC-EVs exhibited a distinct molecular signature primarily driven by the markedly enriched miR-146a-5p, a molecule reported to exert either pro-^[49,50]^ or anti-OA^[51,52]^ effects depending on the biological context. Overall, the miRNA repertoires of BMSC- and ASC-EVs appeared most favorable for OA therapy, characterized by an optimal balance between protective and degradative miRNAs. This profile was supported by the elevated abundance of miR-24-3p, known to inhibit metalloprotease secretion and promote anabolic cartilage metabolism^[53,54]^. The favorable protective-to-destructive ratio was further enhanced by miR-125b-5p, which suppresses inflammatory cytokine and metalloprotease release in OA chondrocytes^[55,56]^, and by miR-222-3p, which inhibits chondrocyte apoptosis and matrix degradation^[57]^. Notably, miR-222-3p expression increases in cartilage subjected to higher mechanical loading, suggesting a role in promoting adaptive remodeling and improving biomechanical resilience^[58]^.

Despite these differences, the comparison between the soluble secretome and EV-associated miRNA profiles revealed overlapping and OA-protective regulatory patterns across all MSC sources. Functionally, this was reflected by the consistent downregulation of key OA-related inflammatory and matrix-degrading genes (*IL-1β*, *IL-6*, *IL-8*, *CTSS*, *CCL5*, and *IDO1*) in human chondrocytes stimulated with IL-1β. In agreement with CD105 expression levels and EV molecular data, ASC- and BMSC-derived secretomes exhibited superior efficacy in suppressing these genes, particularly *IL-1β*^[59]^ and *IDO1*^[60]^, which are strongly implicated in cartilage degradation and inhibition of repair processes. These findings suggest an enhanced capacity of ASC and BMSC secretomes to reprogram inflamed chondrocytes toward a more homeostatic and reparative phenotype. Although the hAMSC secretome also demonstrated anti-inflammatory activity, its comparatively lower efficiency in downregulating *IL-6*, *CTSS*, and *IDO1* expression may limit its standalone therapeutic potential in the context of cartilage homeostasis. Notably, the hAMSC secretome was the only one able to reduce chondrocyte proliferation, an essential process for cartilage repair and ECM synthesis^[61]^, which may partly explain its distinct biological behavior. Nonetheless, a clear protective effect was observed, and the broader impact of hAMSC secretome activity, particularly on immune-cell interactions and joint microenvironment modulation, warrants further investigation.

This study has some limitations. First, the number of assayed factors and miRNAs was limited. Future studies employing broader “omics” approaches, such as mass spectrometry or next-generation sequencing, will be required to capture the full spectrum of secreted components and to refine the overall molecular signature, thereby better reflecting *in vitro* findings and, ultimately, patient outcomes. We opted to test well-described molecules with known functions to ensure biological interpretability and to focus on factors with established relevance to OA pathophysiology, enabling a targeted analysis of their potential therapeutic roles. Furthermore, since EVs were not subjected to RNase treatment, it cannot be excluded that some of the detected miRNAs were externally bound rather than encapsulated within the vesicles. It is also noteworthy that the factors identified in the secretome may exist as soluble molecules, associated with EVs, or both. To provide a comprehensive overview of the protein-associated functional profile, no further distinction between these forms was made. Further studies will help define the impact of factor signatures in the secretomes more clearly. Second, the composition of the MSC secretome may differ between standard culture and serum-free conditions, as serum deprivation can lead to nutrient scarcity. To minimize potential interference between the functional profiles of secretomes obtained under these two conditions, secretome collection was carefully standardized, and bovine origin was excluded. Also, as MSC-derived secretomes are envisioned for use in cell-free therapeutic applications, the presence of serum would not be permissible. Therefore, this well-established serum-free approach was adopted. Third, the *in vitro* assays on chondrocytes provided functional validation of the molecular differences observed across MSC types. However, this simplified system offers only a partial representation of the complex *in vivo* environment. Future studies should employ more relevant models to better capture the biological activity of the secretomes in whole joint tissues and refine the selection of optimal MSC sources and culture conditions prior to translation into clinical applications.

In conclusion, all three MSC sources share a core repertoire of factors with anti-inflammatory and matrix-regulatory properties, providing a consistent protective framework relevant to OA treatment and suggesting a baseline therapeutic potential common to MSCs. However, the differential capacity to modulate OA-related processes appears largely driven by the composition of EV-associated miRNAs, which confer source-specific regulatory effects on chondrocyte behavior and inflammatory gene expression. In this context, BMSC- and ASC-derived EVs displayed the most favorable miRNA signatures, consistent with their superior *in vitro* efficacy in suppressing OA markers and supporting cartilage homeostasis. Conversely, the distinct miRNA profile of hAMSC-derived EVs may underlie their comparatively lower modulatory capacity, despite an overall protective secretory activity. Altogether, these findings highlight the value of systematic EV cargo profiling as a predictive criterion for identifying the most therapeutically competent MSC preparations. Such an approach could accelerate the development of standardized, source-tailored, and clinically translatable MSC-derived products for OA treatment. From a clinical perspective, the results strengthen the rationale for advancing cell-free MSC-based therapies and emphasize the importance of molecular signature-guided selection of the optimal MSC source. Future *in vivo* studies in relevant OA models will be essential to validate these insights and refine MSC product choice, ultimately fostering the translation of personalized, cell-free regenerative strategies that bridge experimental findings with therapeutic application.

## DECLARATIONS

### Availability of data and materials

The datasets generated and/or analyzed during the current study are available in the Open Science Framework repository: https://osf.io/acn4j/?view_only=60ab574adb0743509f35146a405de767.

### AI and AI-assisted tools statement

Artificial intelligence assistance was used in the preparation of this manuscript for language editing.

### Copyright

© The Author(s) 2025.

## References

[1] Kloppenburg M, Namane M, Cicuttini F (2025). Osteoarthritis. Lancet.

[2] Farinelli L, Riccio M, Gigante A, De Francesco F (2024). Pain management strategies in osteoarthritis. Biomedicines.

[3] Kim EH, Jeon S, Park J (2025). Progressing future osteoarthritis treatment toward precision medicine: integrating regenerative medicine, gene therapy and circadian biology. Exp Mol Med.

[4] Zhang H, Felthaus O, Prantl L (2025). Adipose tissue-derived therapies for osteoarthritis: multifaceted mechanisms and clinical prospects. Cells.

[5] Kim GB, Seo MS, Park WT, Lee GW (2020). Bone marrow aspirate concentrate: its uses in osteoarthritis. Int J Mol Sci.

[6] Forbes J, Jackson GR, Knapik DM (2024). The use of amniotic tissue-derived products in orthopedic surgery: a narrative review. Injury.

[7] Sanz-Nogués C, O’Brien T (2021). Current good manufacturing practice considerations for mesenchymal stromal cells as therapeutic agents. Biomater Biosyst.

[8] Di Matteo B, Vandenbulcke F, Vitale ND (2019). Minimally manipulated mesenchymal stem cells for the treatment of knee osteoarthritis: a systematic review of clinical evidence. Stem Cells Int.

[9] Caplan AI (2017). Mesenchymal stem cells: time to change the name!. Stem Cells Transl Med.

[10] Asgarpour K, Shojaei Z, Amiri F (2020). Exosomal microRNAs derived from mesenchymal stem cells: cell-to-cell messages. Cell Commun Signal.

[11] Shimomura K, Wong KL, Saseendar S (2024). Exploring the potential of mesenchymal stem/stromal cell-derived extracellular vesicles as cell-free therapy for osteoarthritis: a narrative review. J Cartil Jt Preserv.

[12] Turlo AJ, Hammond DE, Ramsbottom KA (2023). Mesenchymal stromal cell secretome is affected by tissue source and donor age. Stem Cells.

[13] Dubey NK, Mishra VK, Dubey R, Deng YH, Tsai FC, Deng WP (2018). Revisiting the advances in isolation, characterization and secretome of adipose-derived stromal/stem cells. Int J Mol Sci.

[14] Trzyna A, Banaś-Ząbczyk A (2021). Adipose-derived stem cells secretome and its potential application in “stem cell-free therapy”. Biomolecules.

[15] Kehl D, Generali M, Mallone A (2019). Proteomic analysis of human mesenchymal stromal cell secretomes: a systematic comparison of the angiogenic potential. NPJ Regen Med.

[16] Zhuang J, Hang R, Sun R (2022). Multifunctional exosomes derived from bone marrow stem cells for fulfilled osseointegration. Front Chem.

[17] Ragni E, Papait A, Perucca Orfei C (2021). Amniotic membrane-mesenchymal stromal cells secreted factors and extracellular vesicle-miRNAs: anti-inflammatory and regenerative features for musculoskeletal tissues. Stem Cells Transl Med.

[18] Muntiu A, Papait A, Vincenzoni F (2023). Disclosing the molecular profile of the human amniotic mesenchymal stromal cell secretome by filter-aided sample preparation proteomic characterization. Stem Cell Res Ther.

[19] Zuk PA, Zhu M, Mizuno H (2001). Multilineage cells from human adipose tissue: implications for cell-based therapies. Tissue Eng.

[20] Giordano R, Canesi M, Isalberti M (2014). Autologous mesenchymal stem cell therapy for progressive supranuclear palsy: translation into a phase I controlled, randomized clinical study. J Transl Med.

[21] Papait A, Ragni E, Cargnoni A (2022). Comparison of EV-free fraction, EVs, and total secretome of amniotic mesenchymal stromal cells for their immunomodulatory potential: a translational perspective. Front Immunol.

[22] Szklarczyk D, Kirsch R, Koutrouli M (2023). The STRING database in 2023: protein-protein association networks and functional enrichment analyses for any sequenced genome of interest. Nucleic Acids Res.

[23] Cavalleri T, Angelici L, Favero C (2017). Plasmatic extracellular vesicle microRNAs in malignant pleural mesothelioma and asbestos-exposed subjects suggest a 2-miRNA signature as potential biomarker of disease. PLoS One.

[24] D’haene B, Mestdagh P, Hellemans J, Vandesompele J (2012). miRNA expression profiling: from reference genes to global mean normalization. Methods Mol Biol.

[25] Aparicio-Puerta E, Hirsch P, Schmartz GP, Kern F, Fehlmann T, Keller A (2023). miEAA 2023: updates, new functional microRNA sets and improved enrichment visualizations. Nucleic Acids Res.

[26] Metsalu T, Vilo J (2015). ClustVis: a web tool for visualizing clustering of multivariate data using Principal Component Analysis and heatmap. Nucleic Acids Res.

[27] Iulian Stanciugelu S, Homorogan C, Selaru C (2022). Osteoarthritis and microRNAs: do they provide novel insights into the pathophysiology of this degenerative disorder?. Life.

[28] Endisha H, Rockel J, Jurisica I, Kapoor M (2018). The complex landscape of microRNAs in articular cartilage: biology, pathology, and therapeutic targets. JCI Insight.

[29] Dominici M, Le Blanc K, Mueller I (2006). Minimal criteria for defining multipotent mesenchymal stromal cells. The International Society for Cellular Therapy position statement. Cytotherapy.

[30] Tzavlaki K, Moustakas A (2020). TGF-β signaling. Biomolecules.

[31] Wang W, Rigueur D, Lyons KM (2014). TGFβ signaling in cartilage development and maintenance. Birth Defects Res C Embryo Today.

[32] Fan W, Li J, Wang Y (2016). CD105 promotes chondrogenesis of synovium-derived mesenchymal stem cells through Smad2 signaling. Biochem Biophys Res Commun.

[33] Chang CB, Han SA, Kim EM, Lee S, Seong SC, Lee MC (2013). Chondrogenic potentials of human synovium-derived cells sorted by specific surface markers. Osteoarthritis Cartilage.

[34] Wu CC, Liu FL, Sytwu HK, Tsai CY, Chang DM (2016). CD146+ mesenchymal stem cells display greater therapeutic potential than CD146- cells for treating collagen-induced arthritis in mice. Stem Cell Res Ther.

[35] Li X, Guo W, Zha K (2019). Enrichment of CD146^+^ adipose-derived stem cells in combination with articular cartilage extracellular matrix scaffold promotes cartilage regeneration. Theranostics.

[36] Mukherjee A, Das B (2024). The role of inflammatory mediators and matrix metalloproteinases (MMPs) in the progression of osteoarthritis. Biomater Biosyst.

[37] Wilkinson DJ (2021). Serpins in cartilage and osteoarthritis: what do we know?. Biochem Soc Trans.

[38] Ripmeester EGJ, Caron MMJ, van den Akker GGH (2021). BMP7 reduces the fibrocartilage chondrocyte phenotype. Sci Rep.

[39] Saraiva M, Vieira P, O’Garra A (2020). Biology and therapeutic potential of interleukin-10. J Exp Med.

[40] Al-Qahtani AA, Alhamlan FS, Al-Qahtani AA (2024). Pro-inflammatory and anti-inflammatory interleukins in infectious diseases: a comprehensive review. Trop Med Infect Dis.

[41] (2022). van Helvoort EM, van der Heijden E, van Roon JAG, Eijkelkamp N, Mastbergen SC. The role of interleukin-4 and interleukin-10 in osteoarthritic joint disease: a systematic narrative review. Cartilage.

[42] Arend WP (2002). The balance between IL-1 and IL-1Ra in disease. Cytokine Growth Factor Rev.

[43] Jayasuriya CT, Hu N, Li J (2018). Molecular characterization of mesenchymal stem cells in human osteoarthritis cartilage reveals contribution to the OA phenotype. Sci Rep.

[44] Zhang Y, Liu D, Vithran DTA, Kwabena BR, Xiao W, Li Y (2023). CC chemokines and receptors in osteoarthritis: new insights and potential targets. Arthritis Res Ther.

[45] Reboul P, Guévremont M, Martel-Pelletier J (2003). Hepatocyte growth factor in osteoarthritis: when bone and cartilage decide to have a chat. Arthritis Res Ther.

[46] Bollmann M, Lokau J, Garbers C, Bertrand J (2022). Interleukin-11 - a new cytokine in osteoarthritis?. Osteoarthritis and Cartilage.

[47] Kohrs L, Buettner FFR, Lokau J, Garbers C (2025). The biology of interleukin-6 family cytokines is regulated by glycosylation. Biochem J.

[48] Yan D, Kc R, Chen D, Xiao G, Im HJ (2013). Bovine lactoferricin-induced anti-inflammation is, in part, via up-regulation of interleukin-11 by secondary activation of STAT3 in human articular cartilage. J Biol Chem.

[49] Yang T, Li C, Li Y (2023). MicroRNA-146a-5p alleviates the pathogenesis of osteoarthritis by inhibiting SDF-1/CXCR4-induced chondrocyte autophagy. Int Immunopharmacol.

[50] Qin H, Wang C, He Y (2023). Silencing *miR-146a-5p* protects against injury-induced osteoarthritis in mice. Biomolecules.

[51] Zhang X, Wang C, Zhao J (2017). miR-146a facilitates osteoarthritis by regulating cartilage homeostasis via targeting Camk2d and Ppp3r2. Cell Death Dis.

[52] Zhang H, Zheng W, Li D, Zheng J (2021). miR-146a-5p promotes chondrocyte apoptosis and inhibits autophagy of osteoarthritis by targeting NUMB. Cartilage.

[53] Xu J, Qian X, Ding R (2021). MiR-24-3p attenuates IL-1β-induced chondrocyte injury associated with osteoarthritis by targeting BCL2L12. J Orthop Surg Res.

[54] Philipot D, Guérit D, Platano D (2014). p16INK4a and its regulator miR-24 link senescence and chondrocyte terminal differentiation-associated matrix remodeling in osteoarthritis. Arthritis Res Ther.

[55] Rasheed Z, Rasheed N, Abdulmonem WA, Khan MI (2019). MicroRNA-125b-5p regulates IL-1β induced inflammatory genes via targeting TRAF6-mediated MAPKs and NF-κB signaling in human osteoarthritic chondrocytes. Sci Rep.

[56] Li J, Zhao Z, Chen K (2021). Upregulation of miR-125b-5p relieves chondrocyte inflammation and apoptosis in osteoarthritis by repressing the YAP1/NF-κB pathway. aging.

[57] Song J, Jin EH, Kim D, Kim KY, Chun CH, Jin EJ (2015). MicroRNA-222 regulates MMP-13 via targeting HDAC-4 during osteoarthritis pathogenesis. BBA Clin.

[58] Stadnik PS, Gilbert SJ, Tarn J (2021). Regulation of microRNA-221, -222, -21 and -27 in articular cartilage subjected to abnormal compressive forces. J Physiol.

[59] Vincent TL (2019). IL-1 in osteoarthritis: time for a critical review of the literature. F1000Res.

[60] Alahdal M, Duan L, Ouyang H, Wang D (2020). The role of indoleamine 2,3 dioxygenase 1 in the osteoarthritis. Am J Transl Res.

[61] Chen H, Tan XN, Hu S (2021). Molecular mechanisms of chondrocyte proliferation and differentiation. Front Cell Dev Biol.

